# Pre-service teachers’ empathy and attitudes toward inclusive education—The chain mediating role of teaching motivation and inclusive education efficacy

**DOI:** 10.1371/journal.pone.0321066

**Published:** 2025-04-29

**Authors:** Nana Jiang, Haijun Li, Soon-Yew Ju, Lai-Kuan Kong, Jing Li

**Affiliations:** 1 School of Teacher Education, Heze University, Heze, China; 2 Zhoulou Primary School, Heze, China; 3 Faculty of Administrative Science and Policy Studies, Universiti Teknologi MARA (UiTM) Pahang Branch, Raub, Pahang, Malaysia; 4 Faculty of Business and Management, Universiti Teknologi MARA (UiTM) Pahang Branch, Raub, Pahang, Malaysia; University of Valencia: Universitat de Valencia, SPAIN

## Abstract

Recently, inclusive education has become the direction of special education development. Inclusive education requires that teachers’ educational philosophies and approaches meet the needs of all students. Since pre-service teachers are future educators in training, it is vital to investigate their perspectives on inclusive education. Pre-service teachers’ attitudes, ability and views of inclusive education have an impact on the success of inclusive education practice. However, little literature has examined the influence of psychological mechanism of pre-service teachers toward inclusive education, i.e., the impact of empathy on pre-service teachers’ attitudes toward inclusive education and the mediating effects of teaching motivation and inclusive education efficacy toward the aforementioned relationship. Thus, this study employed Stimulus-Organism-Response’s (S-O-R) model to underpin the research framework that examines the relationship between pre-service teachers’ empathy and attitudes toward inclusive education and the mediating effects of teaching motivation and inclusive education efficacy in the Chinese context. Quantitative data through survey questionnaires was collected from 480 Chinese pre-service teachers and analysed using PLS-SEM. Findings suggested that pre-service teachers’ empathy directly predicted teaching motivation, inclusive education efficacy, and attitudes toward inclusive education. Teaching motivation and inclusive education efficacy together play a chain mediating role in the relationship between empathy and pre-service teachers’ attitudes toward inclusive education. The results of this study offer a theoretical framework that explains in detail the psychological mechanisms underlying the effects of pre-service teachers’ empathy, teaching motivation, and inclusive education efficacy on attitudes towards inclusive education. The research findings also provide practical guidance for the professional development of teachers, talent cultivation, and the development of inclusive education in higher education.

## Introduction

The evolution of special education in the world has transitioned from segregation to inclusion. The 1994 Salamanca Declaration emphasizes that “schools must be inclusive of all children and non-discriminatory.” Everyone has an equal right to education and unique needs in terms of interests, learning, abilities, etc., and schools should accommodate all children, especially those with special educational needs, and meet their specific needs [1]. Inclusive education is a process aimed at reducing educational exclusion by enhancing participation in learning, culture, and community activities. It represents an inclusive educational philosophy that focuses on and responds to the diverse needs of all learners. Furthermore, it is an educational approach that integrates all students, including those with special needs, into a common educational environment [1]. Inclusive education emphasizes equality, individuality, diversity, acceptance, and belonging and advocates the concept of equity in making schools work for all children and promoting access to education for all children. In the endeavor to achieve inclusive education, teachers become the vehicle for implementing and improving the quality of inclusive education, and teachers’ attitudes toward inclusive education directly impact the effectiveness of inclusive education practices [2]. Attitudes toward inclusive education are specific reflections of an individual’s behavioral attitudes toward inclusive education and are expressed in the individual’s cognitive perceptions, affective experiences, and behavioral tendencies toward inclusive education [3]. Teachers with positive attitudes are believed to be more willing to implement inclusive education [4]. Compared to other factors like class size, organisation structure, physical environment, and student background, teachers’ attitudes towards inclusive education play a crucial role in enhancing the quality of inclusive education [5]. In relation to that, it is essential to study pre-service teachers’ attitudes toward inclusive education as they are the prospective teachers at the pre-service stage. The attitudes, concerns, and perceptions of pre-service teachers toward inclusive education not only affect the attitudes of students and parents toward the understanding and acceptance of children with special needs but also influence the success of the practice of inclusive education [6]. Nevertheless, it has been frequently stated that Chinese pre-service teachers have negative or neutral attitudes toward inclusive education [7]. This may have a negative impact on promoting the widespread practice of inclusive education [8]. Therefore, investigating the factors influencing pre-service teachers’ attitudes toward inclusive education and ways to address them has become a common concern in the community in China, as well as the aims of this study. In this study, the term ‘pre-service teachers’ is defined as individuals enrolled in teacher education programs who have not yet completed their studies or graduated.

Pre-service education is an essential stage in the professional development of teachers, the formation of “prospective teachers,” and the initial stage of teachers’ inclusive education literacy [9]. In China, teachers’ inclusive education literacy in the pre-service education stage affects their attitudes and behaviors toward diverse educational targets to a certain extent, ultimately restricting the in-depth development of inclusive education practice and their professional growth. In recent years, pre-service teacher training has been a central topic in teacher education policy, practice, and research [10]. However, literature review indicated that in the field of education, the intrinsic relationship between pre-service Chinese teachers’ empathy and attitudes toward inclusive education has been less explored, and there is a lack of research on the psychological mechanism of interaction among empathy, motivation, efficacy and attitudes, which is worthy of further in-depth exploration. In order to further clarify and validate the relationship between these four variables, this study based on the Stimulus-Organismic-Response (S-O-R) theory, explored the effects and paths of pre-service teachers’ empathy on attitudes toward inclusive education in the Chinese context. The study also examined the intrinsic mechanisms of influence, with the aim of promoting the sustainable development of future teachers.

## Research hypotheses

### Theory underpinning: Stimulus-Organism-Response’s (S-O-R) model

Mehrabian and Russell (1974) had previously suggested that an individual’s behavioral responses (R) are the result of an emotional organism (O) created by environmental stimuli (S) [11]. This study utilized the S-O-R model to demonstrate how teachers’ empathy, as an external stimulus (S), impacts the organism (O) factors, specifically teaching motivation and inclusive education efficacy, which then influence the response (R), namely teachers’ attitudes toward inclusive education. For instance, a teacher who has a high level of empathy might better understand the difficulties faced by students with disabilities, leading to a more inclusive attitude and teaching approach [12]. In the meantime, a teacher who believes in their efficacy in managing a diverse classroom is more likely to adopt inclusive teaching practices and maintain a positive attitude towards inclusion. Motivation, on the other hand, can fuel continuous professional development and persistence in inclusive education, despite the challenges it might present. To sum up, the S-O-R model aids in comprehending how one element affects another, creating a mediated relationship in which motivation is directly impacted by empathy, which in turn drives efficacy that ultimately changes attitudes. This chain mediation highlights the importance of addressing each component in teacher training and development to foster a more inclusive educational environment.

### Empathy and attitudes toward inclusive education

Empathy refers to a person’s ability to perceive the inner world of others [13]. It is a manner of assessing personal feelings and the ability to consider and solve problems from the standpoint of others [14]. Empathy is not only embodied in experiencing the emotions of others but, more importantly, in being able to think from the other person’s point of view to better understand the position and feelings of others. It is one of the fundamental components of emotional experience and plays a crucial role in social interactions [15]. Empathy optimizes inclusive attitudes and relationships with particular groups [16] and reduces prejudice against special groups due to misinformation and negative stereotypes [17]. Empathy for teachers cannot be overstated in the educational field because it is an essential quality for teachers, doctors, and social workers in their interactions with others [18]. It is well known that teaching is a process of social interaction involving students and teachers [19]. Therefore, this process cannot be separated from the existence of empathy, which has an essential worth for professions that require socio-emotional support. Teacher empathy’s cognitive and emotional elements will fully understand students’ emotions through interpersonal interactions [20] and show concern for students through actions [21]. At the same time, empathy helps improve teachers’ teaching effectiveness and promote students’ learning and development [22]. Empathy is a mandatory quality for future teachers. By demonstrating empathy, pre-service teachers can better understand students’ needs and difficulties, improve teachers’ attitudes toward students, and build an excellent teacher-student relationship [23].

Meanwhile, a growing body of literature suggests that empathy is a predictor of positive teacher behaviors in educational contexts and that teacher empathy plays a crucial role in inclusive education in particular [24]. Inclusive education requires that teachers’ educational philosophies and approaches meet the needs of all students. Teacher empathy is a prerequisite if teachers create an inclusive and equitable atmosphere in the classroom [25]. Empathy is at the core of the teaching profession [26] and a critical factor in educational settings [24]. It can increase teachers’ understanding and appreciation of the situation of particular groups [27], which in turn influences teachers’ attitudes toward special groups [28] and, ultimately, their attitudes toward inclusive education. Furthermore, it has been stated that empathy affects teachers’ willingness and teaching motivation [29], subsequently affecting their attitudes towards children with special needs and inclusive education [30]. Based on these arguments, this study proposed the below hypothesis:

**H1**: Empathy has a positive effect on attitudes toward inclusive education.

### Teaching motivation as a mediator between empathy and attitudes toward inclusive education

Teaching motivation is an intrinsic drive for individuals to engage in education, and people with high levels of empathy are more likely to be motivated to engage in education, leading them to be more engaged and passionate about their work [29]. To a certain extent, it can predict teachers’ enthusiasm for the profession and their tendency to improve their professionalism [31]. Teaching motivation includes two dimensions: professional love (PL) and educational interest (EI). Although teaching motivation depends to some extent on personal and background factors [32], among others, research has suggested that empathy, as a personality trait, changes with individual motivation in specific contexts [33]. Recently, studies found that teacher empathy predicts teacher motivation levels [34]. Teaching motivation is an intrinsic drive for individuals to engage in education, and pre-service teachers with high levels of empathy tend to be more motivated to engage in education, prompting them to become more engaged and passionate about it. This is because empathy makes pre-service teachers more concerned about the feelings of students and willing to help special needs students grow. Additionally, as education necessitates establishing an emotional bond with people and comprehending the needs and feelings of students, a person’s willingness to teach itself can also add to their capacity for empathy. Thus, high levels of empathy can stimulate strong teaching motivation. Nevertheless, research demonstrated that motivation contributes to understanding the formation and change of attitudes and is necessary to stimulate or change attitudes [35]. In light of that, it stands to reason that pre-service teachers who are highly motivated to teach will be more positive about their career and the inclusion of special needs students (Groups of students with special educational needs) in regular classrooms. They will also be more willing to practise their profession freely and make significant efforts to raise the standard of education. Based on the foregoing argument, it is hypothesized that:

**H2**: Empathy has a positive effect on teaching motivation.
**H3**: Teaching motivation has a positive effect on attitudes toward inclusive education.
**H4**: Teaching motivation will mediate the relationship between empathy and attitudes toward inclusive education.

### Empathy, inclusive education efficacy, and attitudes toward inclusive education

Inclusive education efficacy may mediate the relationship between empathy and attitudes toward inclusive education. Inclusive education efficacy refers to teachers’ subjective assumptions about their ability to successfully implement inclusive education practices in the context of inclusive education, which includes three dimensions: inclusive teaching efficacy (ITE), collaborative efficacy (CE), and behavior management efficacy (BME) [36]. According to Goleman and Intelligence (1995), the classical theoretical model indicates that emotions are critical constructs related to attitudes [37], and self-efficacy and empathy are two internal elements of social emotions [38]. Previous research has noticed that these two psycho-emotional structures not only play an essential role in the development of teaching effectiveness but also influence teachers’ attitudes toward their students and various dimensions of the educational profession. In addition to positive occupational attitudes, teachers should have socio-emotional competencies to carry out their duties effectively. It was found that among psychosocial competencies, empathy enhances self-efficacy [39], and there is a significant positive correlation between teacher’s empathy and self-efficacy for students with autism spectrum disorders [40]. Empathy and self-efficacy also found to be positively predicted job satisfaction [41]. In the inclusive education context, pre-service teachers with higher self-efficacy are more accommodating of learning-disabled and problem-behavior students and have more positive attitudes toward children with special needs than regular school [42]. Thus, based on the literature review, it is hypothesized that:

**H5**: Empathy has a positive effect on inclusive education efficacy.
**H6**: Inclusive education efficacy has a positive effect on attitudes toward inclusive education.
**H7**: Inclusive education efficacy will mediate the relationship between empathy and attitudes toward inclusive education.

### Chain mediation role of teaching motivation and inclusive education efficacy

Based on the foregoing literature review, empathy may impact attitudes toward inclusive education through two separate mediators: teaching motivation and inclusive education efficacy. When a mediation model contains two or more mediators, a cascading mediation effect is possible if these factors are interrelated [43]. Teachers’ teaching motivation and sense of efficacy are essential factors that influence their attitudes. There is a strong link between teaching motivation and inclusive education efficacy. Pre-service teachers’ motivation to engage in the teaching profession is intrinsic, which can stimulate their interest and enthusiasm for inclusive education work and increase their confidence and self-efficacy in their work. Previous research argued that self-efficacy is related to teachers’ willingness to continue in the profession [44] and that there is a positive correlation between motivation for teaching autonomy and teachers’ self-efficacy in teaching [45]. Therefore, we can conclude that an increase in pre-service teachers’ empathy is associated with increased teaching motivation and a stronger inclusive education efficacy. They are likely to adopt more positive and proactive attitudes towards inclusive education and be more inclined to implement it. Therefore, based on the above findings, the following hypotheses are established:

**H8**: Teaching motivation has a positive effect on inclusive education efficacy.
**H9**: Teaching motivation and inclusive education efficacy together play a chain mediating role in the relationship between empathy and attitudes toward inclusive education.

**Fig 1:**
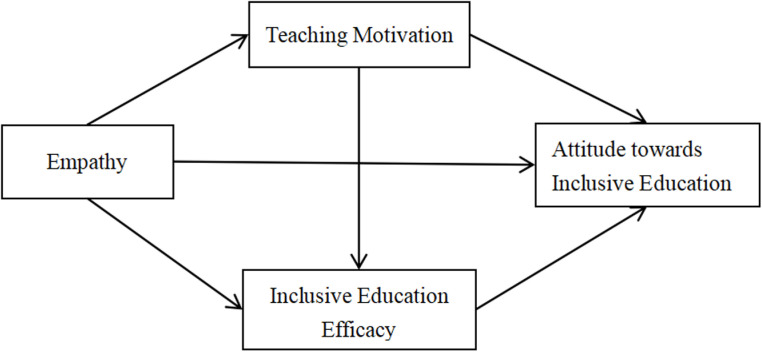
Research framework.

In related to that, the conclusion drawn from pervious literature reviews has been used as groundwork to develop research framework for this study, as showed on Fig 1.

## Materials and methods

This study is a cross-sectional survey conducted using pre-service teachers (18–24 years old) from Heze University, China, as respondents, after obtaining approval from the Ethics Committee of Heze University. The researchers contacted them via WeChat and sent them the link to the questionnaire star. When they opened the link, a consent form message appeared, and they could complete the questionnaire. This research was conducted in accordance with the guidelines of the Declaration of Helsinki. The study was approved by the Ethics Committee of Heze University. The study followed the basic principles of academic ethics, with particular emphasis on the anonymization of the data collected, confidentiality, and non-discrimination of participants.

### Participants

A total of 550 questionnaires were distributed by the researcher, out of which 87.27% (n = 480) were available for data analysis. Among them, the majority are female 84.58% (n = 406) while the rest are male 15.42% (n = 74); 33.54% (n = 161) were first-year students, 32.08% (n = 154) were sophomores, 24.79% (n = 119) were juniors, and remaining 9.59% (n = 46) were seniors. There were 38.75% (n = 186) primary education majors, 27.29%(n = 131) preschool majors, 3.33% (n = 16) psycho-education majors, 18.54% (n = 89) special education majors, 5.84% (n = 28) art education majors, and 6.25% (n = 30) education majors.

### Measurement

#### Empathy.

In this study, empathy refers to the teachers’ ability to understand and share the feelings of students with special need. This study adopted the empathy dimension of Davis’s (1996) Interpersonal Response Indicator Questionnaire [46]. It consists of seven question items on a 7-point scale, ranging from 1 to 7, representing complete disagreement to complete agreement. Teachers used self-evaluations such as “I often feel soft and caring toward those less fortunate than me,” the higher the score on this scale, the higher the level of empathy. The Cronbach’s alpha coefficient for the empathy scale is 0.80 [46].

#### Teaching motivation (TM).

Teaching motivation in this study includes the teachers’ intrinsic and extrinsic motivations to teach, particularly in a challenging and diverse environment. The revised Motivation for Teacher Education Questionnaire by Schepens et al. (2009) was used in this study [47]. The questionnaire consists of four items and two dimensions: professional love (PL) and educational interest (EI), respectively, which indicate the level of an individual’s love for the teaching profession and the level of interest in education. The scale was based on a 6-point Likert scale from 1–6, representing very unconformable to very conformable. The higher the score on the questionnaire, the higher the level of teaching motivation. The Cronbach’s alpha coefficients for the two dimensions of the scale were 0.85 and 0.74, respectively [47].

#### Inclusive education efficacy (IEE).

This study defines inclusive education efficacy as the teachers’ belief in their own ability to effectively teach in an inclusive setting. This study employed the Teachers’ Efficacy of Inclusive Education Practices Scale developed by Sharma, et al. in 2012 [48] and revised by Chinese researcher, Li (2018) [36]. The scale consists of 18 questions and includes three elements: inclusive teaching efficacy (ITE), collaborative efficacy (CE), and behavioral management efficacy (BME). Each factor consists of 6 questions. The scale was based on a 6-point Likert scale from 1–6, representing strongly disagree to strongly agree. The Cronbach’s alpha coefficients for the inclusive education efficacy scale was 0.94 [36].

#### Attitude toward inclusive education (ATIE).

This study used the Scale of Sentiments, Attitudes, and Concerns about Inclusive Education (SACIE-R), a 15-question scale revised by Forlin et al. (2011) [49], to examine the attitudes of the teacher group toward inclusive education in three dimensions. The three dimensions were attitudes (5 questions), concerns (5 questions), and sentiments (5 questions). The scale was based on a 6-point Likert scale from 1–6, representing strongly disagree to strongly agree. The higher questionnaire scores indicating more positive attitudes. The Cronbach’s alpha coefficients for scale was 0.74 [49].

This research questionnaire (Empathy, Teaching motivation & SACIE-R) was initially adapted from an English language version, which was then translated into Chinese by translator (who holds a master’s Degree in Chinese from Heze university) to ease respondents’ comprehension. The study also employed a bilingual English lecturer from Heze university for back translation, to check the accuracy and precision of the translation [50].

### Data analysis

This study used SPSS 29 and Smart PLS 4.1 as tools for statistical data analysis. The data were examined using Smart PLS software by Partial Least Squares Equation Modelling (PLS-SEM). The reason for using Partial Least Squares Regression (PLS) is because it is a commonly used multiple regression method. PLS can simultaneously consider the relationship between the independent, mediator and dependent variables, and it is often more suitable for building predictive models than other methods [51]. In this study, the data sample was analyzed in two steps using Smart PLS software. Firstly, the measurement model was evaluated, and the PLS Algorithm was chosen to test the reliability and validity of the measurement model. Second, the structural model was validated, and bootstrapping and PLS Predict were selected to assess the significance of the path coefficients and the explanatory and predictive power of the model.

## Research results

### Common method bias

In the study of the causal effect of independent variables on dependent variables, there may be a common method bias in observing the association between the two variables when the same measures are utilized [52]. Common method variance (CMV) refers to the use of the same survey instrument that causes erroneous common variance among characteristics, and it is generally present in data collected using self-report scales [53]. The bias introduced by CMV is referred to as common method bias (CMB), a trait-independent systematic error that affects the measure’s validity [54].

In the statistical analysis, the researchers used the full covariance test proposed by Kock (2015) [55]. According to this method, a variance inflation factor (VIF) value less than 3.3 indicates that the model has no multicollinearity problem and the model is well constructed [56]. The VIF values of the present study are shown in Table 1. It can be observed that all the constructs had VIF values below 3.3. Therefore, this study is not affected by CMB.

**Table 1:** Full collinearity testing.

PL	EI	Empathy	ITE	CE	BME	Attitudes	Sentiments	Concerns
1.29	1.29	1.00	1.53	1.51	1.46	1.32	1.27	1.40

Note: PL =  Professional love; EI =  Educational interest; ITE =  Inclusive teaching efficacy; CE =  Collaborative efficacy; BME =  Behavior management efficacy.

### Measurement model

The research model of empathy is a reflective first-order structural model [46]. Teaching motivation (TM) is a reflective second-order structural model, and educational interest (EI) and professional love (PL) are first-order structures [47]. Inclusive education efficacy (IEE) is a reflective higher-order structural model consisting of three first-order structures: inclusive teaching efficacy (ITE), collaborative efficacy (CE), and behavior management efficacy (BME) [48]. Attitudes toward inclusive education is a reflective second-order model, and the three dimensions of attitudes, concerns, and sentiments are first-order reflective structures [49]. In the present study, a two-step procedure was used to assess the measurement model due to the presence of a second-order structure in the current research model [51].

Measurement models were assessed using construct reliability, convergent validity, and discriminant validity methods. Cronbach’s alpha coefficient (CA) was tested to evaluate the reliability of the model constructs. In addition, the composite reliability (CR) test was used to determine the reliability of the model constructs. Following Hair et al. (2019), model construct reliability was established if the values of CA and CR were more significant than 0.70 [51]. Through Table 2, it was showed that these values were over the recommendations of Hair et al. (2019) [51], which confirmed that construct reliability has been established in this study (i.e., ranging from 0.746 to 0.958). Based on the recommendation of Hair et al. (2019), item loadings should be over 0.708 [51]. The loading values of the study items ranged from 0.729 to 0.933, indicating that all remaining items are loaded strongly on their own constructions and less on the other constructs, as shown in Table 2. The findings in Table 2 indicated that the AVE value for all dimensions ranged from 0.609 to 0.860, the minimum acceptable AVE was 0.50 [51], demonstrating a satisfactory degree of convergent validity. The results of the data through the CA and CR values for each dimension in Table 2 below show that PL (0.837, 0.925), EI (0.832, 0.922), TM (0.746, 0.848), Empathy (0.949, 0.958), ITE (0.921, 0.938), CE (0.923, 0.940), BME (0.922, 0.939), IEE (0.750, 0.856), and Attitudes (0.905, 0.929), Sentiments (0.903, 0.928), Concerns (0.905, 0.930), and ATIE (0.779, 0.823). Table 2 shows that the Cronbach’s alpha, outer loading, CA, CR, and AVE values for all study variables meet the suggested threshold. The study investigated the discriminant validity using the heterotrait-monotrait (HTMT) correlation criterion. Discriminant validity was confirmed if the HTMT result was less than or equal to 0.85, as Franke and Sarstedt (2019) recommended [57]. The data from Table 3, the discriminant validity assessment, shows that HTMT is all less than 0.85. Therefore, the discriminant validity of the measurement model meets the criteria.

**Table 2:** Construct reliability and convergent validity assessment.

Construct	Indicator	Factor loadings	Cronbach’s α	CR	AVE
**PL**	PL1	0.926	0.837	0.925	0.860
	PL2	0.928			
**EI**	EI1	0.917	0.832	0.922	0.856
	EI2	0.933			
**TM**	PL	0.827	0.746	0.848	0.737
	EI	0.888			
**Empathy**	Empathy1	0.867	0.949	0.958	0.766
	Empathy2	0.901			
	Empathy3	0.916			
	Empathy4	0.824			
	Empathy5	0.905			
	Empathy6	0.850			
	Empathy7	0.858			
**ITE**	ITE1	0.849	0.921	0.938	0.716
	ITE2	0.850			
	ITE3	0.850			
	ITE4	0.850			
	ITE5	0.839			
	ITE6	0.838			
**CE**	CE1	0.852	0.923	0.940	0.723
	CE2	0.865			
	CE3	0.850			
	CE4	0.857			
	CE5	0.847			
	CE6	0.831			
**BME**	BME1	0.854	0.922	0.939	0.720
	BME2	0.833			
	BME3	0.845			
	BME4	0.846			
	BME5	0.855			
	BME6	0.858			
**IEE**	ITE	0.794	0.750	0.856	0.665
	CE	0.820			
	BME	0.831			
**Attitudes**	Attitudes1	0.847	0.905	0.929	0.724
	Attitudes2	0.850			
	Attitudes3	0.827			
	Attitudes4	0.871			
	Attitudes5	0.860			
**Sentiments**	Sentiments1	0.860	0.903	0.928	0.721
	Sentiments2	0.853			
	Sentiments3	0.855			
	Sentiments4	0.850			
	Sentiments5	0.842			
**Concerns**	Concerns1	0.876	0.905	0.930	0.726
	Concerns2	0.844			
	Concerns3	0.850			
	Concerns4	0.844			
	Concerns5	0.831			
**ATIE**	Attitudes	0.796	0.779	0.823	0.609
	Sentiments	0.729			
	Concerns	0.813			

Note: PL =  Professional love; EI =  Educational interest; TM=Teaching motivation; ITE =  Inclusive teaching efficacy; CE =  Collaborative efficacy; BME =  Behavior management efficacy; IEE =  Inclusive education efficacy; ATIE =  Attitude toward inclusive education.

**Table 3:** Discriminant validity assessment (HTMT).

Construct	BME	CE	EI	ITE	PL	Attitudes	Concerns	Sentiments	Empathy
**BME**									
**CE**	0.525								
**EI**	0.448	0.327							
**ITE**	0.536	0.560	0.208						
**PL**	0.240	0.339	0.571	0.282					
**Attitudes**	0.318	0.396	0.443	0.167	0.409				
**Concerns**	0.501	0.262	0.472	0.252	0.235	0.505			
**Sentiments**	0.227	0.212	0.272	0.408	0.345	0.398	0.469		
**Empathy**	0.209	0.261	0.263	0.246	0.199	0.259	0.262	0.266	

Note: PL =  Professional love; EI =  Educational interest; ITE =  Inclusive teaching efficacy; CE =  Collaborative efficacy; BME =  Behavior management efficacy.

### Structural model

A diagnosis of the covariance of the model has preceded the analysis of the structural model. The data show that the internal VIF values of all constructs are less than 3.3, so there is no colinearity in this study [56]. Following the recommendations made by Hair et al. (2019) [51], a bootstrapping technique with 10000 resamples was used to evaluate the structural models. The standard evaluation criteria include statistical significance and correlation of path coefficients, coefficient of determination (R^2^), assessed effect size (f^2^), and PLS Predict analysis.

**Fig 2:**
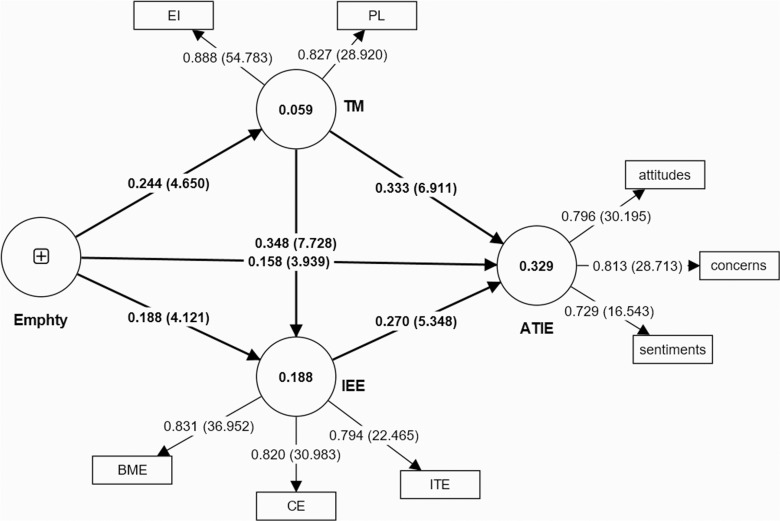
Hypothesis testing.

The results of this study’s direct and indirect hypotheses are shown in Table 4 and Fig 2. This study has six direct hypotheses and three indirect hypotheses. From the results of Table 4, empathy has a positive effect on pre-service teachers’ attitudes toward inclusive education (β = 0.158, t = 3.939, p < 0.01, LL = 0.082, UL = 0.236), empathy has a positive effect on pre-service teachers’ teaching motivation (β=0.244, t = 4.650, p < 0.01, LL = 0.142, UL = 0.343), and empathy has a positive effect on pre-service teachers’ inclusive education efficacy (β=0.188, t = 4.121, p < 0.01, LL = 0.098, UL = 0.281). Thus, H1, H2, and H5 were supported. Teaching motivation has a positive effect on pre-service teachers’ attitudes toward inclusive education (β=0.333, t = 6.911, p < 0.01, LL = 0.239, UL = 0.426), and teaching motivation has a positive effect on pre-service teachers’ inclusive education efficacy (β=0.348, t = 7.728, p < 0.01, LL = 0.255, UL = 0.434). Thus, H3 and H8 were supported. Inclusive education efficacy has a positive effect on pre-service teachers’ attitudes toward inclusive education (β=0.270, t = 5.348, p < 0.01, LL = 0.167, UL = 0.364), thus H6 was supported. Teaching motivation mediates the relationship between empathy and pre-service teachers’ attitudes toward inclusive education (β=0.081, t = 3.756, p < 0.01, LL = 0.023, UL = 0.090), thus H4 was supported. Inclusive education efficacy mediates the relationship between empathy and pre-service teachers’ attitudes toward inclusive education (β=0.051, t = 3.030, p < 0.01, LL = 0.045, UL = 0.132), thus H7 was supported. Teaching motivation and inclusive education efficacy together play a chain mediating role in the relationship between empathy and pre-service teachers’ attitudes toward inclusive education (β=0.023, t = 2.816, p < 0.01, LL = 0.011, UL = 0.042), thus H9 was supported. This indicates that all nine hypotheses were supported. Then, the coefficient of determination (R^2^) was determined based on Chin’s (1998) recommendation that the value should be greater than 0.1 [58]. As shown in Table 4, only Hypothesis 2 had an R^2^ value of less than 0.1, which suggests that empathy has a low explanatory power for teaching motivation. The effect size (f^2^) determines the magnitude of the omitted construct’s influence on a specific endogenous construct, with thresholds of 0.02, 0.15, and 0.35 representing small, medium, and large effects, respectively. As shown in Table 4, the f^2^ values for direct effect hypotheses 1, 2, 3, 5, 6, and 8 all exceed 0.02, indicating a moderate effect [59].

**Table 4:** Hypotheses testing.

Hypothesis	Relationship	β	SE	t	p	LL	UL	R^2^	f^2^	VIF
**H1**	Empathy→ATIE	0.158	0.040	3.939	0.000	0.082	0.236	0.329	0.034	1.107
**H2**	Empathy→TM	0.244	0.052	4.650	0.000	0.142	0.343	0.059	0.064	1.000
**H3**	TM→ATIE	0.333	0.048	6.911	0.000	0.239	0.426		0.137	1.121
**H4**	Empthy→TM→ATIE	0.081	0.022	3.756	0.000	0.023	0.090			
**H5**	Empathy→IEE	0.188	0.046	4.121	0.000	0.098	0.281	0.188	0.041	1.063
**H6**	IEE→ATIE	0.270	0.051	5.348	0.000	0.167	0.364		0.089	1.232
**H7**	Empathy→IEE→ATIE	0.051	0.017	3.030	0.002	0.045	0.132			
**H8**	TM→IEE	0.348	0.045	7.728	0.000	0.255	0.434		0.140	1.063
**H9**	Empathy→TM→IEE→ATIE	0.023	0.008	2.816	0.005	0.011	0.042			

Note: ATIE =  Attitudes toward inclusive education; TM = Teaching motivation; IEE = Inclusive education efficacy.

### PLS-Predict

The PLS prediction procedures were conducted following the methodology proposed by Shmueli et al. (2019) to evaluate the model’s out-of-sample projecting capabilities [60]. The PLS analysis’s root mean squared error (RMSE) was a crucial criterion for assessing predictive performance. This RMSE was compared to the values obtained from a linear model (LM) RMSE, as Danks and Ray (2018) suggested [61]. The expectation was that the PLS analysis would yield lower prediction errors compared to the naïve benchmark (PLS-LM), indicating a medium predictive power. The results in Table 5 reveal that the minority of indicators in the PLS-SEM analysis yield higher prediction errors compared to the naive LM benchmark, thus this indicates a medium predictive power. Furthermore, the data in Table 5 indicates that all Q^2^ values are higher than 0, suggesting sufficient predictive relevance.

**Table 5:** PLS-predict.

Indicators	PLS-SEM_RMSE	LM_RMSE	PLS-LM	Q^2^ predict
**PL1**	1.146	1.153	-0.007	0.021
**PL2**	1.129	1.142	-0.013	0.024
**EI1**	1.101	1.112	-0.011	0.034
**EI2**	1.119	1.127	-0.008	0.051
**BME1**	1.141	1.155	-0.014	0.020
**BME2**	1.095	1.108	-0.013	0.030
**BME3**	1.115	1.127	-0.012	0.014
**BME4**	1.105	1.121	-0.016	0.031
**BME5**	1.100	1.112	-0.012	0.028
**BME6**	1.128	1.142	-0.014	0.022
**CE1**	1.110	1.124	-0.014	0.043
**CE2**	1.131	1.144	-0.013	0.039
**CE3**	1.085	1.097	-0.012	0.034
**CE4**	1.118	1.133	-0.015	0.050
**CE5**	1.118	1.132	-0.014	0.036
**CE6**	1.079	1.087	-0.008	0.037
**ITE1**	1.144	1.161	-0.017	0.040
**ITE2**	1.106	1.124	-0.018	0.028
**ITE3**	1.126	1.142	-0.016	0.043
**ITE4**	1.122	1.138	-0.016	0.026
**ITE5**	1.106	1.116	-0.010	0.043
**ITE6**	1.087	1.101	-0.014	0.028
**Attitudes1**	1.094	1.103	-0.009	0.030
**Attitudes2**	1.095	1.110	-0.015	0.051
**Attitudes3**	1.070	1.075	-0.005	0.028
**Attitudes4**	1.096	1.101	-0.005	0.043
**Attitudes5**	1.106	1.117	-0.011	0.040
**Concerns1**	1.146	1.144	0.002	0.048
**Concerns2**	1.041	1.041	0.000	0.032
**Concerns3**	1.091	1.100	-0.009	0.050
**Concerns4**	1.075	1.076	-0.001	0.045
**Concerns5**	1.049	1.050	-0.001	0.020
**Sentiments1**	1.083	1.089	-0.006	0.023
**Sentiments2**	1.111	1.120	-0.009	0.040
**Sentiments3**	1.033	1.036	-0.003	0.051
**Sentiments4**	1.088	1.095	-0.007	0.052
**Sentiments5**	1.097	1.106	-0.009	0.038

Note: PL =  Professional love; EI =  Educational interest; ITE =  Inclusive teaching efficacy; CE =  Collaborative efficacy; BME =  Behavior management efficacy.

## Discussion

Teachers’ positive attitudes toward inclusive education are vital to achieving inclusive education in schools. Recently, researchers have started to consider improving pre-service teachers’ attitudes toward inclusive education from an empathic perspective [62]. Therefore, this study examined the link between pre-service teachers’ empathy and attitudes toward inclusive education and the chain mediating role of teaching motivation and inclusive education efficacy in the Chinese culture. The research findings indicated that pre-service teachers’ empathy directly predicted teaching motivation, inclusive education efficacy, and attitudes toward inclusive education; pre-service teachers’ empathy also influenced attitudes toward inclusive education through two mediating variables: teaching motivation and inclusive education efficacy. Teaching motivation and inclusive education efficacy together play a chain mediating role in the relationship between empathy and pre-service teachers’ attitudes toward inclusive education.

### Relationship between empathy and attitude toward inclusive education

The study’s findings indicate that pre-service teachers’ empathy has a positive effect on their own attitudes toward inclusive education. This suggests that pre-service teachers with higher levels of empathy are more likely to have positive attitudes toward inclusive education. This finding validates research hypothesis 1 and is consistent with the previous finding [28]. Empathy, the ability to share and understand the feelings and thoughts of others, is one of the fundamental components of emotional experience and plays a crucial role in social interactions [15]. Empathy is a crucial factor that influences a person’s capability to engage in interpersonal and emotional communication, and the more empathetic an individual is, the more he or she can put himself or herself in another person’s position and understand their thoughts and feelings. Strongly empathetic pre-service teachers can comprehend the challenges and requirements of children with special needs from the standpoint of children with special needs and are, therefore, more likely to accept special needs children and be willing to contribute to the development of these children [63]. In conclusion, the level of pre-service teachers’ empathy positively predicts attitudes toward inclusive education. Pre-service teachers’ empathy increases understanding and knowledge of the situation of special needs students [27], which in turn influences attitudes toward special needs children and inclusive education. Therefore, by developing pre-service teachers’ empathy toward special needs, students can improve their attitudes toward these groups [62] and increase teachers’ output of positive behaviors toward children [27].

### Mediating effect of teaching motivation between empathy and attitudes toward inclusive education

The results of this study indicated that pre-service teachers’ empathy has a positive effect on teaching motivation, and teaching motivation has a positive effect on attitudes toward inclusive education, while teaching motivation mediates the effect between empathy and attitudes toward inclusive education. This suggests that pre-service teachers’ empathy indirectly affects attitudes toward inclusive education through the bridge of teaching motivation. The results of this study validated research hypotheses 2, 3, and 4. This study supports the previous findings [29,64,65]. Research indicates that individuals with higher levels of empathy are more capable of understanding others’ situations and emotions and thus feel more socially responsible and actively engage in social activities that promote social justice [66]. Similarly, pre-service teachers who are more empathetic will be able to understand and relate to the situation and feelings of children with special needs, and this empathy will make them realize their responsibilities as future teachers toward children with special needs. This empathy makes them aware of their responsibility as future teachers towards children with special needs. This sense of responsibility motivates them to have a stronger intention and belief in teaching, to be more willing to accept students with special needs, to pay attention to the interests of students with special needs, and to hope that students with special needs can have access to fair educational opportunities and quality educational resources.

### Mediating effect of inclusive education efficacy between empathy and attitudes towards inclusive education

The results of this study indicate that pre-service teachers’ empathy has a positive effect on inclusive education efficacy, that inclusive education efficacy has a positive effect on attitudes toward inclusive education. Meanwhile, inclusive education efficacy has a mediating effect between empathy and attitudes toward inclusive education. These findings suggest that pre-service teachers’ empathy also indirectly influences attitudes toward inclusive education through the bridge of inclusive education efficacy. These findings validated the research hypotheses 5, 6, and 7 and also aligned with the previous findings [67–69]. The mechanism of action is that teachers with empathy are more confident in their ability to perform the tasks and work of teaching practice in inclusive education, which can directly affect teachers’ attitudes toward inclusive education [70].

### Chain-mediating effect of teaching motivation and inclusive education efficacy between teachers’ empathy and attitudes toward inclusive education

The results of this study suggested that pre-service teachers’ teaching motivation has a positive effect on inclusive education efficacy, and teaching motivation and inclusive education efficacy together play a chain mediating role in the relationship between empathy and pre-service teachers’ attitudes toward inclusive education. These findings suggested that pre-service teachers’ empathy affects attitudes toward inclusive education through the chain mediation of “teaching motivation and inclusive education efficacy.” Empathy also indirectly enhances attitudes toward inclusive education through the chain mediating effect of teaching motivation and inclusive education efficacy. The results of this study validated research hypotheses 8 and 9 and also aligned with the previous findings [67,69]. Empathy, teaching motivation, and inclusive education efficacy are all significantly and positively predicted attitudes toward inclusive education that the stronger the empathy, teaching motivation, and inclusive education efficacy, the higher the degree of pre-service teachers’ attitudes toward inclusive education. These insights suggesting that teachers’ benevolence, intention to engage in the teaching profession, and sense of efficacy not only directly affect teachers’ educational behaviors, but also indirectly affect educational behaviors by influencing the attitudes toward inclusive education. The mechanism of action lies in the fact that empathy can enhance teachers’ willingness and motivation to engage in education, augment their sense of efficacy in addressing the diverse learning needs of all students in inclusive classrooms, and subsequently foster a more perceptive and accepting attitude towards students with special needs.

### Significance and limitations of the study

Educators are the critical deliverers of education, and the training of pre-service teachers is the prerequisite and foundation for future teacher-building and education quality improvement. Therefore, this study has few specific theoretical and practical significances.

This study has important theoretical contributions. First of all, although there have been previous studies on the effect of empathy on attitudes toward inclusive education, the research on the effect of pre-service teachers’ empathy on attitudes toward inclusive education is still immature in China. This study explores the influence mechanism of empathy on attitudes toward inclusive education and broadens the understanding of the relationship between affection and attitudes. Secondly, there are few studies on empathy and inclusive education efficacy in the previous literature. This study reveals the positive predictive effect of empathy on inclusive education efficacy and enriches the literature on the relationship between empathy and inclusive education efficacy. Moreover, it also discusses the mediating effect of inclusive education efficacy between empathy and attitudes toward inclusive education, which provides a new research perspective on the concept of inclusive education efficacy. Lastly and most importantly, this study constructs a chain mediation model of teaching motivation and inclusive education efficacy between empathy and attitudes toward inclusive education and provides a more thorough understanding of how empathy affects attitudes toward inclusive education.

This study has important practical significance. First, this study found that empathy has a positive and significant effect toward pre-service teachers’ attitudes toward inclusive education. Therefore, for higher education institutions and educational organizations, it is possible to enhance pre-service teachers’ attitudes towards inclusive education by fostering pre-service teachers’ affective dimensions. Secondly, this study found that empathy promotes pre-service teachers’ attitudes toward inclusive education through the chain mediation effect of teaching motivation and inclusive education efficacy. This finds a deep inner enhancement path for pre-service teachers’ attitudes toward inclusive education. Based on this, higher education institutions and educators should pay attention to the cultivation of pre-service teachers’ teaching motivation and the formation of inclusive education efficacy, care about pre-service teachers’ psychological growth and emotional needs, and stimulate pre-service teachers’ motivation and initiative toward inclusive education. It satisfies pre-service teachers’ intrinsic pursuit of a sense of significance and achievement toward inclusive education, to ultimately promote the generation of teachers’ attitudes toward inclusive education.

Reflecting on the whole research process, it was found that there were some limitations. The main limitations are as follows: this study used cross-sectional data and further longitudinal studies or experiments should be adopted in the future. This study only evaluates attitudes towards inclusive education from the perspective of individual pre-service teachers. Future research could examine the effects of empathy toward attitudes toward inclusive education from both pre-service and in-service teachers’ perspectives. Regarding the relationship between empathy and attitudes toward inclusive education, there may be other influencing factors, moderating variables, and mediating variables. Future researchers can pay more attention to the positive effects of empathy and explore another ways to enhance attitudes toward inclusive education.

## Conclusions

In conclusion, this study reveals the link between pre-service teachers’ empathy and attitudes toward inclusive education. It verifies the respective independent mediating role and chain mediating effect of teaching motivation and a sense of inclusive education efficacy. Educators must recognize the importance of empathy in inclusive education, enhance pre-service teachers’ teaching motivation, and inclusive education efficacy to improve attitudes toward inclusive education and build a harmonious and inclusive educational atmosphere. This provides essential information and theoretical foundations for interventions to enhance the quality of pre-service teacher training and inclusive education in China.

## Supporting information

S1 Data Minimum data set. (XLSX)
